# The Treatment of Typhoid Excreta

**Published:** 1902-10-18

**Authors:** 


### THE TREATMENT OF TYPHOID EXCRETA.

We are often inclined to wonder how far medical journalism represents medicine, and to question whether what appears in medical papers is not sometimes written by journalists whose ideas are up in the clouds and who have but little practical acquaintance with what is going on in daily life in this far too imperfect world of ours. Commenting on a paper by Dr. Veeder of Lyons, New York State, in which the author had insisted that typhoid fever is not spread by water alone, and that an undisinfected typhoid bacillus when thrown out upon the soil will be likely to spread contagion, the *Post-*

*Graduate* affects a certain surprise that such an obvious statement should be regarded as worthy of promulgation, and pretends that not only is this view thoroughly accepted by the profession, which it doubtless is to a large extent, but that it is equally thoroughly acted upon, which it as certainly is not. The *Post-Graduate* says "Raw vegetables grown in such a soil and eaten may convey the bacillus to the stomach, but does not every up-to-date physician know this, and does he not always disinfect typhoid excretions? Is it possible that there are any regimental surgeons in our army, or in an army of any civilised nation, that does not disinfect even doubtful stools before burying them in the trenches? Let us go further. Is there any family or consulting physician in the land that neglects the disinfection of typhoid stools? If there be many such, much elementary work remains to be done in the profession. We had hoped that this was everywhere done."

Now it is all very well for the *Post-Graduate* to make believe that principles and practice are so closely allied that the fact that it is known that the infective particles in typhoid excreta can be deprived of their infective properties by heat, or by certain somewhat elaborate chemical methods, of necessity and as a matter of course involves the further fact that all such excreta are so dealt with. But we ask how far is this actually done in real life, and what machinery there is for doing it? Do country practitioners, either here or in America, habitually order, or superintend, or give instruction in the processes necessary for the disinfection of excreta? Do the sanitary officials supply the apparatus or the chemicals necessary for a complete and satisfactory sterilisation not only of typhoid but of all doubtful excreta? (We do not mean the supply of a little pink powder to scatter over them, but such an amount and quality of disinfectant as to thoroughly kill the bacilli). Does the practitioner in a town even advise such a course, and does he not rather urge that everything shall be as quickly as possible washed down the drain? Are army medical officers, either here or in America, provided with the means of having excreta sterilised? Nay, we would even ask are the excreta of typhoid fever patients in our great civil hospitals thoroughly disinfected in the sense of being sterilised? If these things are not done, why make believe?

